# Reduced diversity in human mitogenomes from Eivissa (Balearic Islands)

**DOI:** 10.3389/fgene.2026.1852529

**Published:** 2026-06-25

**Authors:** Laura Clara Verdesca, Julen Aizpurua-Iraola, Elisa Marí-Cardona, Maria Barber-Olives, Marc Tormo, Raquel Rasal, Núria Bonet, Vanessa Villalba-Mouco, Francesc Calafell

**Affiliations:** 1 Institut de Biologia Evolutiva (CSIC-UPF), Universitat Pompeu Fabra, Barcelona, Spain; 2 Departament de Medicina i Ciències de la Vida (MELIS), Universitat Pompeu Fabra, Barcelona, Spain; 3 Clinical Trials Office, Institut d’Oncologia Vall d’Hebron (VHIO), Barcelona, Spain; 4 Departament de Medicina i Ciències de la Vida (MELIS), Genomic Core Facility, Universitat Pompeu Fabra, Barcelona, Spain; 5 Departament de Medicina i Ciències de la Vida (MELIS), Scientific Computing Core Facility, Universitat Pompeu Fabra, Barcelona, Spain

**Keywords:** Eivissa, haplgroup L2c, human isolated populations, Menorca, mitogenome, mtDNA

## Abstract

The Balearic Islands, off the NE coast of the Iberian Peninsula, have a rich and diverse history. We had previously reported that genomic diversity in one of the Balearics, Eivissa, shows the signs of a reduced effective population size and founder effect when compared not only to populations of the mainland but also to Menorca, another Balearic Island of comparable area and population size but with a more open historic trajectory. In the current work, we investigate whether the same pattern can be observed in the mitogenomes, and leverage the rich phylogeographic information contained in mitochondrial DNA (mtDNA) to detect the sources of the maternal lineages in Eivissa and Menorca. Indeed, Eivissa shows a reduced mtDNA haplotype diversity compared to Menorca and mainland populations, but similar to that in islands in other archipelagos (Canaries, Orkney, Shetland). However, other measures of diversity, such as number of segregating sites or nucleotide diversity do not show this reduction, probably because one of the founder lineages in Eivissa belongs to the deeply divergent L2c haplogroup, which is of Sub-Saharan origin and with a time to the most recent common ancestor that suggests that it was introduced during the Punic colonization of the island. Still, direct comparison with ancient DNA samples of various periods did not yield any sequence match that could indicate continuity with the population present in the islands before the Catalan-Aragonese conquest of the 13th century.

## Introduction

The Balearic archipelago, off the NE coast of the Iberian Peninsula, comprises four inhabited islands: Menorca (Minorca), Mallorca, Eivissa (Ibiza) and Formentera; the latter two are often referred to as the Pityusic Islands. Eivissa and Menorca have similar areas (600–700 sq. Km) and current populations (100,000–150,000), but slightly different historical trajectories. The archipelago was first colonized in the second half of the 3rd millennium cal. BCE (calibrated Before Common Era), probably by people from the Iberian Peninsula; while the prehistory of Mallorca and Menorca is characterized by the Talaiòtic period, with abundant examples of cyclopean architecture, these structures are rare in Eivissa and Formentera. However, the eponymous Eivissa town (Ebussus) was funded by the Phoenician in 654 BCE and fell later into the Punic (Carthaginian) sphere (Aubet, 2001). After the fall of the Roman Empire, the islands were briefly occupied by the Vandals, later to be regained by the Byzantines. In 903 CE, the islands were formally annexed to the Cordova Caliphate. In the following three centuries, the Islamic religion and the Arabic culture were prevalent in the islands; this period ended along the 13th century, when the Balearics were conquered by the Kingdom of Aragon (Mallorca in 1,230, Eivissa in 1,235, but Minorca was made into a fiefdom and not formally annexed until 1,287). The Balearics were settled with mostly Catalan colonists, which introduced the Catalan language and culture that have persisted to this day. The islands, together with Roussillon, constituted the Kingdom of Mallorca in 1,276, but reverted to the crown of Aragon in 1,349. Thereafter, the Balearics have followed the vicissitudes of the kingdoms of Aragon and then Spain, except for Minorca, which, given the strategic location of the port of Maó (Mahon) changed hands a number of times between Spain, Great Britain and France along the 18th century, to be reannexed to Spain in 1802. The British left their mark in the local economy, transport and a few words in the local dialect, but their demographic contribution may have been negligible (Aizpurua-Iraola et al., 2025).

Several papers have contributed to the genetic characterization of the human population of the Balearic Islands (including the Chueta crypto-Jews of Mallorca), using blood groups and other polymorphisms (Picornell et al., 1991; Picornell et al., 1992; Picornell et al., 1996; Picornell et al., 1997), control region mtDNA sequences (Picornell et al., 2005; Ferragut et al., 2020), Y-chromosome polymorphisms (Tomàs et al., 2006; Rodríguez et al., 2009; Ferragut et al., 2020; Navarro-López et al., 2024), and X-chromosome markers (Ferragut et al., 2015; Ferragut et al., 2017; Ferragut et al., 2021). A study based on a genome-wide SNP array revealed that Eivissa is an outlier in the Western European genetic landscape (Biagini et al., 2019); comparison with ancient DNA allowed to reject that this differentiation was due to the Phoenician or Punic background of the island (Zalloua et al., 2018; Biagini et al., 2019; Ringbauer et al., 2025). Instead, runs of homozygosity and estimates of effective population size pointed to intense genetic drift as the source of the Eivissan differentiation (Biagini et al., 2019). In a follow-up study, Menorca was added as a control, and exomes were sequenced to gauge the impact on mutation load of Eivissan isolation (Aizpurua-Iraola et al., 2025); the results were that Menorca showed far fewer genetic signs of isolation, as expected from its history, but the Eivissan population did not carry a higher frequency of deleterious mutations. Recently (Ventayol-Guirado et al., 2025), the incidence of transthyretin amyloidosis, an autosomal dominant Mendelian disease, was found to be significantly lower in Eivissa than in Menorca or Mallorca. A recent aDNA study of a medieval Islamic cemetery in Eivissa (Rodríguez-Varela et al., 2026) revealed that the ancestry of the local population at that time was admixed between European and North African sources, with two individuals of fully Sahelian ancestry. Since the current population of Eivissa shows a much smaller proportion of North African ancestry (Aizpurua-Iraola et al., 2025), this may point to a subsequent population replacement, likely linked to the Catalano-Aragonese conquest.

Mitochondrial DNA has a number of properties that can complement a genome-wide view of genetic diversity. Its lack of recombination and well-established phylogeography (van Oven et al., 2014) allow to pinpoint the broad geographical origin of each mitogenome. Currently, over 60,000 human whole mitogenomes, comprising >40,000 haplotypes, are readily available for analysis (Huber et al., 2025), which means that perfect mitogenome matches within and between populations imply a recent connection between their bearers. A previous survey of mtDNA, based solely on hypervariable region I, revealed lower diversity and the effect of genetic drift in Eivissa, but not in Mallorca or Minorca (Picornell et al., 2005).

We have analysed the complete mitogenomes of 15 volunteers from Eivissa (plus 18 previously published sequences (Zalloua et al., 2018) and 24 from Menorca, in order to characterize their internal diversity and describe the phylogeographic origins of their haplotypes, in these two populations that are geographically close, but with different histories embedded in their genomic diversity.

## Materials and methods

### Samples and sequencing

We collected and sequenced 15 Eivissan and 24 Menorcan samples obtained from healthy unrelated volunteers with the condition of having their four grandparents born in the respective island. The participants signed their written informed consent after being appropriately informed about the aims of the project. This project was approved by the CEIm-PSMAR IRB in Barcelona (2019/8900/I). All the research in this manuscript has been conducted following the principles outlined in the Declaration of Helsinki.

DNA was extracted using the QIAamp DNA blood mini kit (Qiagen GmbH, Hilden, Germany) following the manufacturer’s recommendations and was quantified using Quantifiler^TM^ Human DNA Kit on 7500 SDS Reak-Time PCR System (Applied Biosystems). PCR amplifications were performed in four different fragments as in Aizpurua-Iraola et al. (2022). NexteraXT libraries were prepared and sequenced in a Miseq sequencer (Illumina) following the Illumina mtDNA Genome Guidelines (EMEA, 2025).

### Sequence processing

Sequences were processed according to the GATK best practices protocol (Van der Auwera et al., 2013). First, an initial quality check was performed using FastQC (Babraham Bioinformatics, 2025). Then, the raw sequencing reads were mapped against the revised Cambridge Reference Sequence (rCRS) (Andrews et al., 1999) using the BWA-MEM algorithm (Li, 2013). The PCR duplicates were removed with Picard Tools (2025), base quality scores were recalibrated with GATK’s Base Quality Score Recalibration (BQSR) (McKenna et al., 2010), and a final quality report was obtained with Qualimap2 (Okonechnikov et al., 2016). Finally, the sequence variants were called with GATK tools HaplotypeCaller and GenotypeGVCFs (McKenna et al., 2010).

Sequences were manually inspected for insertions and deletions (indels, particularly in the 303–315 and 16,184–16193 stretches) and heteroplasmic sites with Integrative Genomics Viewer (IGV) v. 2.12.1 (Robinson et al., 2011). A site was called as heteroplasmic when none of the variant calls was present in >90% of the reads; in subsequent analyses, the majority allele was used in heteroplasmic sites. Average coverage per site and per individual were estimated with samtools (Li et al., 2009).

### Reference dataset

We added 18 published Eivissan mitogenomes (Zalloua et al., 2018), for a total dataset of 33 samples from Eivissa and 24 from Menorca. As a reference dataset, we reviewed Genbank and the literature, and gathered population samples (of size ≥30) from Europe, the Middle East and North Africa, *autonomous communities* (first-order administrative divisions) in Spain, and several islands and archipelagos (Sardinia, the Canaries, Orkney, Shetland). See the complete list, sample sizes and references in Supplementary Table 1.

Ancient mitogenomes from a Punic site in Eivissa (Zalloua et al., 2018) (n = 9) and from several sites and periods in Mallorca and Menorca (Villalba-Mouco et al., submitted.), were also used as reference. Of the latter, only sequences without any missing nucleotide calls were retained, resulting in n = 10 sequences from Mallorca and n = 63 from Menorca. For population distance analyses, and given their large sample size, ancient Menorcan sequences were arbitrarily grouped into pre-Roman (n = 36, mostly Bronze and Iron Age), Roman and Byzantine (n = 11), and Medieval (n = 20, mostly Islamic).

### Statistical analysis

Haplogroups were determined with Haplogrep v.3.2.1 (Schönherr et al., 2023) using the forensic update of phylotree v.17 (Dür et al., 2021). Basic diversity parameters and pairwise difference distances (*ϕ*_*ST*_) were computed with Arlequin v. 3.5 (Excoffier and Lischer, 2010). Multidimensional scaling was computed with the *isoMDS* function of the *MASS* R package. Subsampling (without replacement) was performed with the *sample* function in R, and subsequently diversity parameters in 1,000 resampled sets were computed with the corresponding functions in the *ape* and *pegas* R packages. Times to the most recent common ancestor were estimated with ρ (Saillard et al., 2000) and a mutation rate of 2.74 x 10^−8^ substitutions per nucleotide per year (2,203 years per mutation in the whole mitogenome) (Posth et al., 2016) and with BEAST 1.10 (Suchard et al., 2018); the Extended Bayesian Skyline model implemented in this program was also used to estimate the time trajectory of effective population sizes (Ne) in Eivissa, Menorca, and in 100 subsamples of size 30 drawn from Catalonia. For the BEAST analysis, the substitution model we used was the HKY with 4 gamma categories for rate distribution and invariant sites, as indicated by jModelTest2 (Darriba et al., 2012) and set a strict clock model assuming all tree branches evolve at the same rate. We used UPGMA as the starting trees for the analysis, which was conducted in five independent runs with 25,000,000 iterations, sampling the result every 25,000 iterations. The resulting log files were combined using LogCombiner and the combined log file was checked with Tracer 1.7 (Rambaut et al., 2018) to ensure effective sample size (ESS) values over 200 for every parameter.

## Results

### Eivissan and Menorcan sequence diversity

**FIGURE 1:**
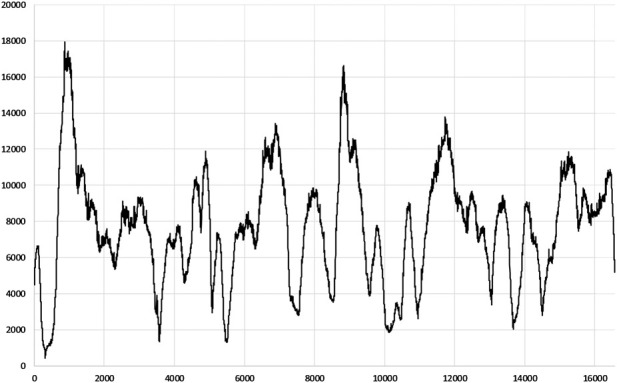
Average sequencing coverage along the mitogenome

We sequenced 15 new mitogenomes from Eivissan volunteers and 24 from Menorca; the former were analyzed jointly with 18 previously published mitogenomes (Zalloua et al., 2018). Average coverage per sample was 7,818X (range 2,653–12,068X), and, although some fluctuations were observed along the sequence, the minimum coverage per position was 442X (at position 310) (Figure 1).

The full haplotypes obtained and their inferred haplogroups are shown in Supplementary Table 2. Out of 33 sequences in Eivissa, we found 31 different haplotypes, while all sequences in Menorca were different from one another. However, length polymorphisms at the 303–315 and 16,184–16193 stretches, heteroplasmic sites, and the hypermutable 16,519 position, while clearly useful for personal identification in a forensic context, are not sufficiently stable to provide meaningful comparisons within and between populations in a historical context. Therefore, in all subsequent analyses, we have omitted the variants in these sites. Taking this into consideration, the number of different haplotypes drops to 25 in Eivissa and 22 in Menorca, and one of these (belonging to haplogroup L2c, discussed below) is shared between Eivissa and Menorca. Haplotype diversity is 0.983 and 0.993 respectively. The number of segregating sites is 213 in Eivissa and 233 in Minorca; the average pairwise differences are respectively 35.24 and 34.35.

In order to place the sequence diversity in Eivissa and Menorca in a wider geographical context, we gathered the population sequence data sets available for Europe, West Asia and North Africa, with an emphasis on islands and isolated populations (see Methods for details). Diversity measures for 50 additional populations can be found in Supplementary Table 1. However, direct comparisons for these statistics are not possible, since sample sizes in Eivissa and Menorca are clearly smaller than many of the reference populations. Then, we downsampled reference populations to match sample sizes in Eivissa and Menorca.

**FIGURE 2:**
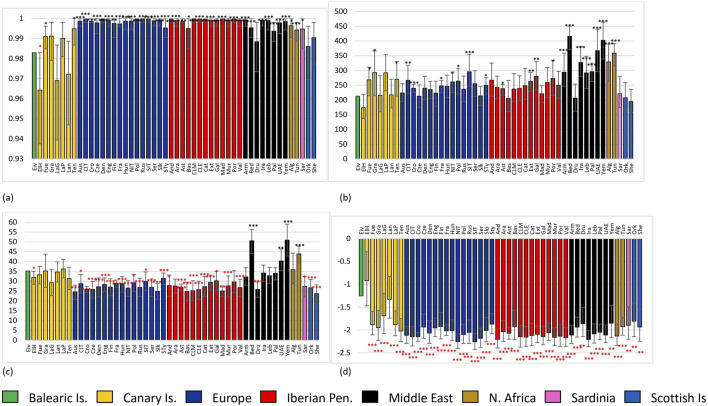
Internal diversity parameters in Eivissa and reference populations. **(a)** haplotype diversity; **(b)** number of polymorphic sites; **(c)** mean pairwise differences; **(d)** Tajima’s D. See population abbreviations in Supplementary Table 1. Error bars indicate the central 2.5% and 97.5% percentiles of the distribution obtained by subsampling to n = 33 (Eivissa’s sample size). Stars indicate the position of Eivissa’s values in each population’s distribution. No stars imply that Eivissa’s values fall between the 2.5% and 97.5% percentile. One black star: between the 0.25% and 2.5% percentiles; two black stars: between the 0.1% and 0.25% percentiles; three black stars: below the 0.1% percentile. One red star: between the 97.5% and 99.75% percentiles; two red stars: between the 99.75% and 99.9% percentiles; three red stars: above the 99.9% percentile.

**FIGURE 3:**
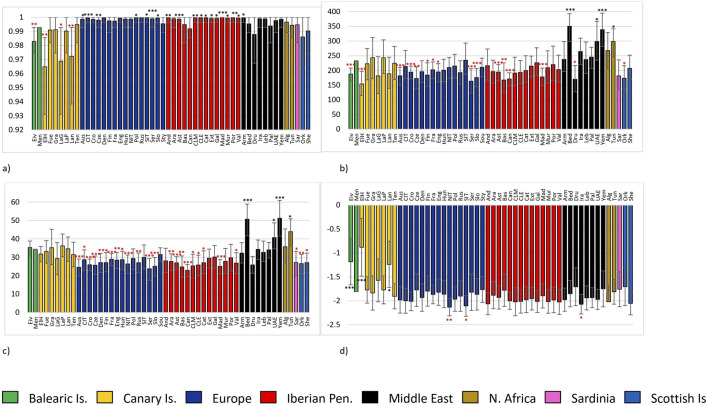
Internal diversity parameters in Menorca and reference populations. **(a)** haplotype diversity; **(b)** number of polymorphic sites; **(c)** mean pairwise differences; **(d)** Tajima’s D. See population abbreviations in Supplementary Table 1. Error bars indicate the central 2.5% and 97.5% percentiles of the distribution obtained by subsampling to n = 24 (Menorca’s sample size). Stars indicate the position of Menorca’s values in each population’s distribution. No stars imply that Menorca’s values fall between the 2.5% and 97.5% percentile. One black star: between the 0.25% and 2.5% percentiles; two black stars: between the 0.1% and 0.25% percentiles; three black stars: below the 0.1% percentile. One red star: between the 97.5% and 99.75% percentiles; two red stars: between the 99.75% and 99.9% percentiles; three red stars: above the 99.9% percentile.

Haplotype diversity in Eivissa is clearly smaller than in almost all populations of the reference dataset (Supplementary Tables 1, 3; Figure 2a), once downsampled. It falls within the central 95% of the empirical distribution only for most of the Canary Islands, Shetland and Orkney, and the isolated Druze in Lebanon. It is higher only than in El Hierro, the most isolated of the Canaries. On the contrary, haplotype diversity in Menorca (Supplementary Tables 1, 4; Figure 3a) is closer to the values in many of the reference populations, and is significantly higher than in Eivissa and three of the Canaries (El Hierro, La Gomera, and Lanzarote).

In spite of the lower haplotype diversity, Eivissan sequences show, once corrected for sample size, a number of polymorphic sites that is closer to those in the reference populations, falling in the central 95% of the distribution in 24/49 reference populations (Supplementary Tables 1, 3; Figure 2b), and below in the remaining 25 reference populations. The number of polymorphic sites in Menorca is higher than in Eivissa, and even higher than in 15 Iberian and European reference populations, while it is lower than in Armenian, Emirati, Yemeni, and Tunisian (Supplementary Tables 1, 4; Figure 3b).

Mean pairwise differences show a striking pattern in Eivissa, since they are higher than in many European populations (Supplementary Tables 1, 3; Figure 2c), although they are similar or lower than in some North African and Middle Eastern populations (Bedouin, Emirati, Yemeni, Tunisian; see Figure 2c); a similar pattern was found in Menorca (Supplementary Tables 1, 4; Figure 3c). In fact, mean pairwise differences within each population are strongly correlated with the frequency of the deeply divergent L haplogroups (r^2^ = 0.7181, p ≈ 10^−15^, Pearson’s correlation coefficient).

We also computed Tajima’s D primarily as a way to compare S to π in the different populations. Tajima’s was clearly higher (though still negative) in Eivissa compared to the rest of the reference dataset (Supplementary Tables 1, 3; Figure 2d), being above the 97.5% percentile in all populations but in two of the Canary Islands: El Hierro and Lanzarote. On the contrary, Tajima’s D in Menorca fell within the 2.5% and 97.5% percentiles of the subsample distributions in all but a few populations: Eivissa, El Hierro and Lanzarote (below their respective 2.5% percentiles) and Northern Italy, Southern Italy and Iran (above their 97.5% percentiles).

Overall, the pattern of internal sequence diversity in Eivissa seems to be dominated by a few lineages, resulting in a lower haplotype diversity and fewer segregating sites, but, since some of these lineages (and particularly one belonging to L2c, discussed below) are highly divergent, this has caused the higher mean pairwise difference and Tajima’s D seen in Eivissa. On the contrary, patterns of internal diversity in Menorca (Supplementary Tables 1, 4; Figure 3d) are similar to those in the European populations of the reference dataset, implying a higher number of founders and/or less intense drift than in Eivissa.

**FIGURE 4:**
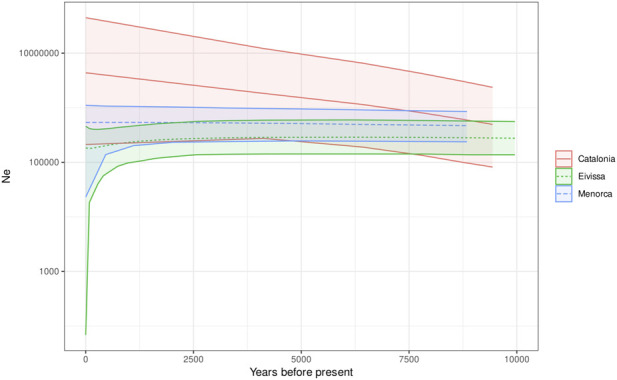
Estimated Ne time trajectories for Eivissa, Menorca and Catalonia; the latter is an average of 100 subsamples of size 30. The median and the 95% HPD are indicated for each population.

We used BEAST to estimate the effective population size (Ne) trajectory for Eivissa, Menorca and Catalonia (see Methods). Currently, Eivissa exhibits the smallest estimated Ne (Figure 4). The estimate for Menorca was 2.93 times that of Eivissa, and the estimate for Catalonia is nearly 24 times larger. While Catalonia demonstrates a linear increase of Ne over time, Menorca and Eivissa show much flatter trajectories, with the latter even displaying a slight decrease in recent times.

### Eivissan and Menorcan mitogenomes in a phylogeographic context

Overall, six haplotypes are shared with other populations (Supplementary Tables 5). One of those, found in 14 individuals of at least five different populations (mostly from N. Europe) is at the root of haplogroup H1+16,189, is probably polyphyletic and therefore of little phylogeographic relevance. Four other haplotypes (in haplogroups H1e2, T2a1a, K1a13a and T2b25), carried by five samples from Eivissa and two from Menorca, are shared solely with Spanish samples. Finally, one haplotype carried by two U5a1i1 samples from Eivissa is shared with one individual each from Scotland, Sweden and Norway.

We can now widen our scope and consider more distant matches by describing the worldwide population frequencies of the haplogroups found in Eivissa and Menorca (Supplementary Table 6-8). The most frequent haplogroups in Eivissa (12.1% each) are T2a1a and L2c. The former is quite rare, and it is found at frequencies 0.4%–0.5% in the Caribbean, Central Asia, Central Europe and the United States (Supplementary Table 7), and at slightly lower frequencies (0.3%–0.4%) in Eastern Europe and Spain and Portugal. Haplogroups derived from T2a1a (T2a1a1 through T2a1a7) are also rare and mostly prevalent in Western and Central Europe, and in the United States Therefore, a precise geographical origin cannot be pinpointed for these four identical sequences in Eivissa, other than a match with a Spanish sample of unknown origin (Supplementary Table 5).

**FIGURE 5:**
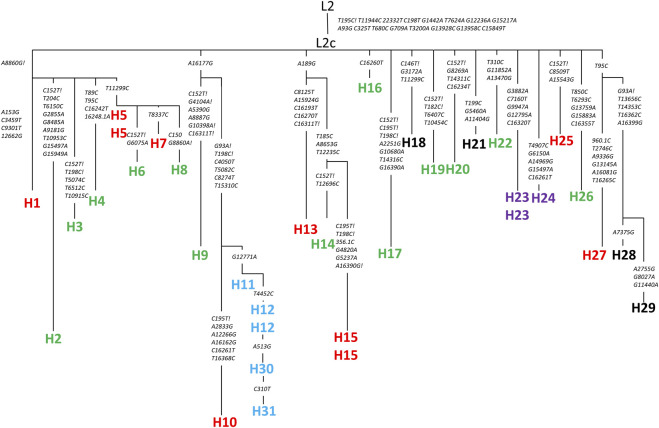
Maximum parsimony tree of the L2c paragroup sequences. Haplotype label colors indicate their geographical origin: Blue, Balearic Islands; red, the Americas; green, Africa; purple, Europe; black, unknown. See Suppl. Table 9 for details on the origins and references of each sequence.

The L2c haplogroup is represented by four samples in Eivissa and one in Menorca, which carry four different sequences (the Menorcan haplotype matches one in Eivissa). L2c is relatively common in West Africa (2.4%, Supplementary Table 7), and is found also in countries were Africans were trafficked into (Brazil, 0.45%; Caribbean, 0.46%; United States, 0.18%); it is rare in Spain and Portugal (0.05%), and in SW Europe (0.03%), while, to the best of our knowledge, has not been described elsewhere. It was not found in Punic (Zalloua et al., 2018; Ringbauer et al., 2025) or Islamic (Rodríguez-Varela et al., 2026) sites in Eivissa, but, in a sample of 50 individuals from Eivissa in which 404 bp of the HVRI were sequenced, three carried the same HVRI sequence as our L2c individuals (Picornell et al., 2005). Within the L2c phylogeny (Figure 5; see the references for each sequence in Supplementary Table 9), the Eivissan sequences form a clade that is distantly related to an African-American sequence. The nucleotide differences among the Balearic sequences allow to estimate a TMRCA of 3,100 ya (sd, 1,800; computed with ρ, see Methods); a Bayesian estimate with BEAST yields 2,348 ya, with a highest 95% posterior density interval of 444–5344 ya. The current geographic distribution of L2c, as well as its phylogenetic structure, point to West Africa as a possible origin of this haplogroup. It is tempting to speculate that L2c may have arrived in Eivissa with the Arabic domination of the island. In fact, ancient DNA analysis of remnants found in Islamic cemeteries in Eivissa revealed two individuals whose ancestry was completely from West Africa, although they did not carry L2c (Rodríguez-Varela et al., 2026). However, the TMRCA of Eivissan L2c predates the Arabic conquest, and the arrival of L2c into Eivissa may have coincided with the first stable settlement of the island by the Punic/Phoenician, although, as stated above, L2c was not found in 17 ancient mitogenomes from Punic sites in Eivissa (Zalloua et al., 2018; Ringbauer et al., 2025). The presence of L2c in Minorca was caused by much more recent migration: although born in Menorca, the maternal grandmother of the Minorcan volunteer carrying L2c bore typically Eivissan surnames.

A haplogroup with a clear geographical origin is M1a1 in Menorca (4.2%). It is found in NE Africa at 1.8% (Supplementary Table 6), and in neighboring regions (Arabia, E Africa, and the Levant) at smaller frequencies. A plausible hypothesis is that it was brought to Menorca by the Medieval Arabic rulers, although other possibilities, such as the Romans or the Byzantines, or even earlier contacts, cannot be ruled out.

Most other haplogroups found in Eivissa and Menorca have a general West Eurasian distribution without a clear focus. One can just point to K1a4a1 (12.5%) in Eivissa, which is most prevalent in the Iberian Peninsula and NW Africa, a similar distribution to that of the V paragroup (3% in Eivissa).

### Eivissa and Menorca in the mitogenome diversity landscape

**FIGURE 6:**
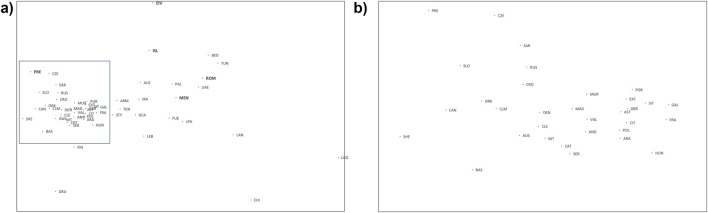
Multidimensional scaling plot of the *ϕ*_*ST*_ distance matrix in **(a)** the reference dataset (Yemen excluded) with ancient DNA samples included; **(b)** blown-up inset. Population abbreviations as in Supplementary Table 1.

*ϕ*_*ST*_ genetic distances between Eivissa, Menorca and the population reference dataset can be found at Supplementary Table 10. Both islands are quite distant from most other populations, particularly in the case of Eivissa. Therefore, while the average distance between any two populations is 0.0291, Eivissa averages 0.0571. The smallest distances from Eivissa (all, though, >0.033) are with a few Middle Eastern and North African populations, as well as with South Tyrol, La Palma (in the Canary Islands) and Menorca. The average distance from Menorca to any population in the reference dataset is 0.0286, and it is closest to South Tyrol, Tunisia and UAE (*ϕ*_*ST*_ < 0.01). Multidimensional scaling was applied to the *ϕ*_*ST*_ distance matrix (Supplementary Table 1). Since the plot was dominated by the outlier position of the Yemen population, we excluded it from the analysis (Figure 6). Eivissa occupies a peripheral position in the plot, but notice that other small, isolated populations can also be found as outliers, such as La Gomera (Canary Islands), the Druze, and Shetland. Menorca plots closer to Palestine, Algeria, and the Canary Islands of Fuerteventura and La Palma. These populations, and, in general, those found towards the positive end of the X-axis in the plot do not seem to share specific haplogroups but have rather low frequencies of the H haplogroup. The frequency of the H haplogroup is 12.5% in Menorca, while in the Iberian populations is 44.6% on average (range 36.2%–52.3%). This low frequency of H in Menorca had already been found in an article based on the hypervariable region I (24%, N = 46) (Picornell et al., 2005), and therefore cannot be attributed solely to the smaller sample size of our study (Rodríguez-Varela et al., 2026). report the haplogroups of 13 ancient samples from a Medieval Islamic cemetery in Eivissa; only one of these haplogroups, namely, HV0+195 is shared with the modern sample. However, this is a broadly distributed haplogroup, with 87 instances found in Genbank, originating from Western Europe and North Africa.

### The historical trajectory of mitogenome diversity in Eivissa and Menorca

Some ancient complete mitogenomes are available for Eivissa and Menorca, which can give a glimpse on the possible continuity of maternal lineages through time. For Eivissa, nine mitogenomes where obtained from Punic sites (Zalloua et al., 2018). Of those, no sequences matched the current diversity, and only one presented the same haplogroup that can now be found. This was T2b, which was carried by one Punic individual who did not bear any additional mutation from the root of the haplogroup, and one modern individual, which carried additionally T8277C, C13150T, and C16184T. Therefore, this cannot be taken as strong evidence for continuity. Given the absence of any matches in a sample n = 9, the binomial probability distribution can be used to estimate a joint maximum frequency for current sequences in the aDNA pool. The probability of not observing current sequences in a sample of size 9 would be p = (1-f)^9^, where f is the ancient frequency of current sequences. Equating arbitrarily p to 0.05, one can derive that the maximum estimated of the current sequences in the aDNA pool would be f = 1-exp (ln (0.05)/9) = 0.2831.

As for Menorca, a larger number of ancient sequences are available, ranging from the Pre-Talaiòtic (Bronze Age) to Islamic periods (8th-13th centuries CE). Again, no sequences were shared between ancient and modern Menorca, and only one haplogroup was shared, namely, the paragroup H. Applying the same formula as above, no sequence matches implies that the current mitogenome pool would have a maximum frequency of 0.046 in ancient Menorca. Given the sample sizes and the quality of the sequences, we could group them in three broad groups (Pre-Roman, Roman/Byzantine, and Islamic; see Methods), and compute *ϕ*_*ST*_ distances. Distances between modern and ancient Menorcans were 0.056, 0.0009 and 0.025 for the Pre-Roman, Roman/Byzantine, and Islamic pools, respectively. The fact that modern Menorcans appear so close to Romans and Byzantines may indicate the effects of the Islamic expulsion after the Christian conquest, but it should be interpreted with caution, given that this is the smallest (N = 11) of the three samples.

## Discussion

We have analyzed 33 complete mitogenomes from Eivissa and 24 from Menorca. We have found in both populations, but more pointedly in Eivissa, the footprints of founder effect and reduced effective population size. This is also reflected in the autosomal genomes of both islands and may be a consequence of the traditionally smaller population size in Eivissa, and island with a more arid climate and poorer agriculture than Menorca. Eivissa shows a clear reduction in haplotype diversity, but this reduction is not so extreme in the number of polymorphic sites, and, actually, mean pairwise differences (or π) are higher in Eivissa than in any other European population in our reference database. This pattern is likely due to the fact that one the founder lineages in Eivissa (that is, a mitogenome sequence found at high frequency in Eivissa but absent elsewhere) belongs to haplogroup L2c, which is highly divergent from the rest of the sequences in Eivissa (and, in general, from the mtDNA sequences that are prevalent in Europe). This haplogroup was also found in a volunteer from Menorca, but whose maternal grandmother, born also in Menorca, carried a maternal surname of Eivissan origin. This is an additional dimension of the reduced effective population size in Eivissa: native Eivissan surnames are relatively few and most of them are quite characteristic of the island (Biagini et al., 2019). A direct, Bayesian estimate of female Ne showed reduced values in Eivissa when compared to Menorca or Catalonia; note that, although probably reliable in relative terms, the absolute values derived by BEAST are unplausible, since they are actually higher than the current census population. The reduced mtDNA Ne agrees with the patterns observed in genowide autosomal diversity (Biagini et al., 2019). Actually, the Eivissan differentiation is more extreme in mtDNA: while, in a PCA for genomewide autosomal SNPs, Eivissa plots within the European cluster (Biagini et al., 2019; Aizpurua-Iraola et al., 2025), in an mtDNA-based MDS, Eivissa is an outlier within Europe (Figure 6a). This is expected, given that, in general, mtDNA has ∼1/4 of the autosomal Ne.

Menorca shows also a reduction in haplotype diversity (although to a lesser extent than Eivissa), but a slightly higher number of polymorphic sites, and, as Eivissa, a higher mean nucleotide pairwise difference; while higher than the estimate for Eivissa, Menorca’s Ne is still one order of magnitude smaller than that for Catalonia. Again, this pattern matches that observed for genowide autosomal variation, where Menorca showed a slight reduction in Ne when compared to Iberian populations. One notorious feature in the mtDNA phylogeographic landscape of Menorca is the reduced frequency of haplogroup H, which was also observed in a larger sample of HVI sequences (Picornell et al., 2005). Since haplogroup H is most frequent in Western Europe and its frequency declines towards the East and South (Achilli et al., 2004), its low frequency in Menorca may be interpreted as a sign of a genetic contribution from North Africa or the Levant. However, the relative frequencies of non-H haplogroups in Menorca are similar to those in Iberia and Western Europe, as measured with Euclidean distance over the frequencies of non-H haplogroups, (Supplementary Table 11). Therefore, the low frequency of haplogroup H in Menorca may also be the result of genetic drift.

In our reference dataset, we included a number of island and isolated populations: the seven Canary Islands, Sardinia, the Orkney, Shetland, South Tyrol and the Druze (see Supplementary Table 1 for references). A distinctive trait shared by many of these populations (all but Sardinia and South Tyrol) is a reduced haplotype diversity compared to mainland populations (Supplementary Table 1, 3; Figure 2a). However, they do not share other measures of genetic diversity reduction: they tend to have a number of segregating sites or mean pairwise differences that are on a par or higher than mainland populations. In fact, these measures correlate with the presence among the founder lineages of deeply divergent sequences belonging to haplogroups L0 through L6, which are more frequent in Sub-Saharan Africa.

As references, we have also used ancient DNA sequences from Eivissa and Menorca. For the former, sample size was reduced, and the absence of matching sequences between the ancient and the modern samples should not be taken as evidence of lack of historical continuity. In Menorca, however, sample sizes are larger and allow population comparisons by historical period. Still, modern and ancient samples did not share any sequence, and even terminal haplogroup matches were few and involving haplogroups that are rather common in Western Europe. The MDS plot shows Menorcans from different periods occupying very different positions, but the usual caveats about aDNA degradation and sampling biases apply in this case. Therefore, we did not find direct evidence for historical continuity of maternal lineages, which would point to population replacement associated with the 13th-century Catalan conquest of the islands. However, L2c, the second most frequent haplogroup in Eivissa, with a TMRCA of 2,000–3,000 years ago and absent in the mainland, could indicate that a lineage introduced in Punic or Roman times has persisted to the present day, although it has yet to be found in the aDNA record.

In summary, we have found that the mtDNA diversity patterns in Eivissa and Menorca mirror those in the autosomal genome, presenting evidence of reduced effective population size in Eivissa, and, to a lesser extent, in Menorca. The main caveat to this conclusion is the reduced sample sizes we could obtain; however, by subsampling sequences in the populations of the reference dataset, we could not only make meaningful comparisons, but also provide empirical statistical distributions and tests for differences in diversity parameters.

## Data Availability

The datasets generated for this study have been deposited in Genbank; Accession Numbers are listed in Supplementary Table 2.
